# Interplay of p62-mTORC1 and EGFR signaling promotes cisplatin resistance in oral cancer

**DOI:** 10.1016/j.heliyon.2024.e28406

**Published:** 2024-03-21

**Authors:** Hsiu-Chuan Chang, Cheng-Chieh Yang, Lai-Keng Loi, Chi-Hsun Hung, Cheng-Hsien Wu, Yu-Cheng Lin

**Affiliations:** a Department of Dentistry, School of Dentistry, National Yang Ming Chiao Tung University, Taipei, Taiwan; b Institute of Oral Biology, School of Dentistry, National Yang Ming Chiao Tung University, Taipei, Taiwan; c Department of Stomatology, Oral & Maxillofacial Surgery, Taipei Veterans General Hospital, Taipei, Taiwan; d Oral Medicine Innovation Center (OMIC), National Yang Ming Chiao Tung University, Taipei, Taiwan

**Keywords:** OSCC, p62, mTOR, EGFR, Cisplatin resistance

## Abstract

Cisplatin resistance poses a major challenge in the treatment of oral squamous cell carcinoma (OSCC). Deeper investigations into the mechanisms underlying this drug resistance is of great importance. Here, we used cellular assays and clinical immunohistochemistry to examine molecular pathways involved in both innate and acquired cisplatin resistance. We demonstrated that the p62-mTORC1 signaling complex plays a pivotal role, and is driven by the EGFR signaling network, specifically through the PI3K-Akt axis and the transcription factor C/EBP-β. Elevated *p*-mTOR expression was associated with cancer relapse and poor prognosis among oral cancer patients. Additionally, we illustrated that mTOR inhibitors enhance the cytotoxic effect of cisplatin, by employing cancer stem cell characteristics. Our work unveils fundamental mechanisms for cisplatin resistance, thereby presenting therapeutic implications for OSCC.

## Highlights

• In OSCC, the p62-mTORC1 signaling is pivotal in intrinsic tolerance, as well as transition to acquired resistance to CDDP.
• The p62-mTORC1 signaling is facilitated by the EGFR-PI3K-Akt-C/EBP-β axis.
• Elevated mTORC1 activity correlates with a higher recurrence rate in advanced OSCC patients.

## Graphical abstract

**Figure:**
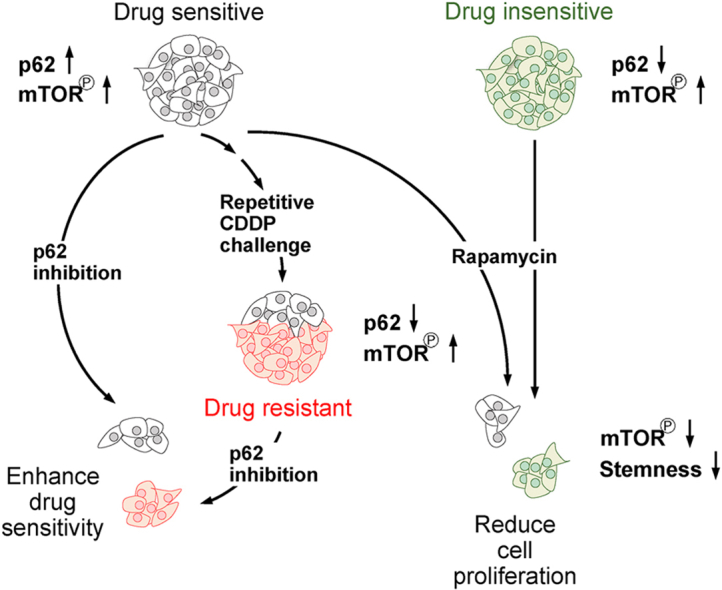

## 1 Introduction

Oral squamous cell carcinoma (OSCC) is the major type of head and neck squamous cell carcinoma (HNSCC) leading to worldwide annual incidence of more than 50,000 cases and a disease-related mortality greater than 50% [1,2]. Despite advances in targeted therapies and surgical interventions, the overall survival (OS) rate of oral cancer patients has shown only marginal improvement over the years [[3], [4], [5]]. One of the major obstacles in the effective treatment of OSCC is the development of resistance to chemotherapy agents, particularly cisplatin (CDDP).

CDDP-based agent is the first-line chemotherapeutic drug for the treatment of multiple types of solid tumors, including bladder, esophageal, ovarian, and head and neck squamous cell carcinoma (HNSCC) [6,7]. Chemotherapeutic interventions often encounter intrinsic or acquired drug resistance via multiple molecular mechanisms. Substantial research efforts have revealed the molecular mechanisms for the CDDP resistance, including activation of various survival pathways, hindrance of drug binding to the genetic targets, and outflow or inactivation of drug [8]. These mechanisms were thought to be induced by stochastic genetic mutations occurring within a tumor cell population during drug exposure, conferring selected clones with drug resistance [9]. However, more recent findings have reported non-mutational mechanisms of drug resistance [10], such as the existence of a small fraction of drug refractory cancer stem cells [11], epigenetic mechanisms [12], and the emergence of drug tolerant persisters (DTPs) [[13], [14], [15]], and the activation of epidermal growth factor receptor (EGFR) signaling network.

The complex web of EGFR signaling drives three main pathways: extracellular signal-regulated kinase 1 and 2 (ERK1/2), phosphatidylinositol 3-kinase/protein kinase B (PI3K/Akt), and signal transducer and activator of transcription 3 (STAT3) [16]. Although EGFR signaling has been implicated in CDDP resistance in various cancer types including non-small cell lung cancer (NSCLC) [17], cervical cancer [18], and OSCC [19], it remains unclear which specific pathway predominantly contributes to the drug resistance in oral cancer.

In addition to the role of EGFR in cancer biology, the mechanistic target of rapamycin (mTOR) signaling is a major pathway that mediate a wide variety of cellular physiological processes, such as apoptosis, autophagy, amino acid metabolism, and proliferation [20]. Further, emerging evidence has suggested that mTOR signaling contributes to drug resistance in melanoma, breast cancer, lung cancer, and head and neck cancer [[21], [22], [23], [24]]. Intriguingly, mTOR activities mediated by insulin-like growth factor 1 (IGF-1) presented conflicting results on the drug resistance in different cancer types [10,12]. Central to the mTOR signaling pathway is the multifunctional scaffold protein, p62, essential for the formation of the mTOR complex 1 (mTORC1) [25]. While p62 is a passive biomarker for autophagy state, it also serves as a scaffold stimulator of mTORC1 at the lysosomal membrane in normal physiological conditions [21].

Given these observations, we aimed at exploring a critical yet underrepresented crosstalk between the EGFR signaling network and the p62-mTORC1 complex in the case of CDDP resistance in oral cancer. We specifically examined the role of transcription factor C/EBP-β (CCAAT enhancer binding protein β) [26]. Additionally, we investigated the relationship between mTOR activation and cancer stem cell (CSC) characteristics, given that CSC is thought to be a driving factor for chemoresistance and cancer relapse [27]. By using both cellular assays and immunohistochemical data from patient specimens, we sought to elucidate relevant molecular mechanisms underpinning CDDP resistance, thereby contributing to the development of more effective therapeutic interventions for oral cancer.

## 2 Materials and methods

### 2.1 Cell culture, reagents, drug inhibitors, and phenotypic analysis

All OSCC (SAS, OECM-1, and OC4) and HNSCC (FaDu and HSC3) cell lines utilized in this study underwent genetic authentication via STR DNA typing and routine mycoplasma contamination testing using the EZ-PCR Mycoplasma Test Kit (Biological Industries, Israel) to ensure consistent cell identity. The SAS (RRID:CVCL_1675; JCRB No.: JCRB0260), OECM-1 (RRID:CVCL_6782), FaDu (RRID:CVCL_1218; ATCC No.: HTB-43), and HSC3 (RRID:CVCL_1288; JCRB No.: JCRB0623) cell lines were acquired from either the American Type Culture Collection (ATCC, USA) or Japanese Collection of Research Bioresources Cell Bank (JCRB Cell Bank, Japan), or derived as per previously published protocols [28]. The OC4 cell line, a human tongue cell carcinoma, was established in our lab from primary cells and has been continuously passaged for over 100 generations [28]. All cultivation conditions are described in Table S1. Insulin (10 μM) and IGF-1 (100 ng/ml) were used to treat cells for 48 h. EGF (100 ng/ml) stimulated EGFR signaling for 0.5–24 h. Doses of cisplatin (cis-Diammineplatinum(II) dichloride; CDDP) were 5 μM or 10 μM, and the dose of Taxol was 20 nM, respectively. Before cisplatin or EGF treatments, cells were pretreated for 1 h with inhibitors AG1478, LY294002, U0126, and S3I-201, all purchased from Merck Millipore (USA), at 10 μM (or 20 μM). To produce cells with transient knockdown, pilot tests confirmed 60 nM and 100 nM as the effective concentrations over 24 and 48 h for the sip62 and siC/EBP-β oligonucleotide (Santa Cruz Biotechnology, USA), respectively, using siCtrl as a parallel control. TransFectin Lipid Reagent (BioRad, USA) was used for all transient expression experiments. For cell viability determined by the MTT assay, cells were plated on 96-well or 48-well plates at densities of 1500 or 3000 cells/well, in replicates of at least six, and allowed to adhere for 24 h. Treatments included various doses of cisplatin (0–20 μM) for 48 h, IGF-1 (100 ng/ml), Rapamycin (100 nM), and Metformin (10 mM) individually over 4 days, or combining cisplatin with either Rapamycin (50 nM) or Metformin (2 mM) for 48 h or over a period of 4 days. DMSO was a control. For growth curve using the trypan-blue exclusion assay, cells were plated on 24-well plates (5000 cells/well) in triplicate and treated with cisplatin, IGF-1, Rapamycin, and Metformin post 24-h adhesion. Measurements were taken over the next 4–5 days with DMSO as a control. For cell survival determined by the clonogenic assay (also known as the colony formation assay), cells were pre-treated with cisplatin and then re-seeded onto 6-well plates (1500 cells/cm^2^) in triplicate. During the siRNA treatment period, cells were treated either with or without the drug. Nine days or more later, colonies were washed, stained with 0.5% crystal violet diluted in either methanol or ethanol, and counted (diameter greater than 500 or 1000 μm) using microscopy. For ALDH activity assay, cells were treated with IGF-1, Rapamycin, and Metformin for 48 h. After treatment, cells were exposed to bodipy-labeled aminoacetaldehyde (BAAA, 1 μg per 1,000,000 cells; ALDEFLUOR Kit; Stem Cell Technologies, USA), which serves as a fluorescent non-toxic substrate for ALDH, and incubated. An aliquot from each sample was treated with diethylaminobenzaldehyde (DEAB, 30 nM; ALDEFLUOR Kit), as an ALDH-specific inhibitor for negative control. Analysis used a flow cytometer (FACSCalibur; BD Biosciences, USA) and FlowJo software (BD Biosciences). Unless otherwise specified, all other reagents were purchased from Sigma-Aldrich (USA).

### 2.2 Generation of CDDP-induced resistant cell subpopulations

SAS cells were treated with dose-escalating cisplatin to derive CDDP-induced resistant cell subpopulations. Cells (250,000 cells/6-cm plate) were treated with 0.1, 0.25, 0.5, and 1 μM cisplatin for 3 days, respectively. Survivors from the 1 μM treatments were re-plated at 500,000 cells/10-cm plate and treated with 1.5 μM cisplatin for 7 days or more. During this period, they were passaged, and the fresh drug-containing medium was replaced every 2 days until survival rates went below 20%. Then, the drug was withdrawn, with a drug-free medium introduced every 3 days until clones appeared. The clones achieved approximately 80% confluency per plate after a drug-free period of 20 days. Isolated clones were continuously cultivated and expanded in increasing doses for three rounds (5, 10, and 15 μM), with each treatment lasting 7 days. Clones adhering post the maximum dose (15 μM) were deemed drug-induced resistant cell subpopulations and cryogenically stored. Before analysis, the resistant strain was thawed and recovered in drug-free medium first, followed by further drug rechallenging studies or determinations of the IC50 values.

### 2.3 Generation of stable knockdown cell subpopulations

Stable knockdown cells were produced using lentiviruses given by Dr. Kuo-Wei Chang. A lentivirus vector (pLKO.1-puro; RNA Technology Platform and Gene Manipulation Core of Academia Sinica, Taiwan) carried sequences for expressing either short hairpin p62 (shp62) or short hairpin luciferase (shLuc; Control). These vectors, combined with packaging plasmids (pMD.G and pCMVΔR8.91), were transfected into 293T cells to generate lentiviruses. The infected cells underwent puromycin selection for 7 days, yielding a subpopulation with stable knockdown of p62.

### 2.4 Western blot analysis

Cells were lysed using NP-40 Lysis Buffer containing 50 mM Tris-HCl (pH 7.5; Amresco, USA), 150 mM NaCl (Amresco), 1 % (v/v) sodium deoxycholate, 1% (v/v) NP-40, 1X EDTA-free Protease Inhibitor Cocktail (Roche, USA), 25 mM sodium fluoride, 5 mM sodium orthovanadate, and 25 μg/ml aprotinin, then centrifuged to obtain supernatants. Protein concentrations were measured using the Bradford assay (Bio-Rad Protein Assay Kit; Bio-Rad, USA). 60 μg of lysates underwent sodium dodecyl sulfate polyacrylamide gel electrophoresis and were transferred to nitrocellulose membranes (Merck Millipore). Membranes were blocked using 2% bovine serum albumin or 5% nonfat dry milk in phosphate buffered saline containing 1% (v/v) Tween 20, incubated overnight with primary antibodies, then with horseradish peroxidase-conjugated secondary antibodies for 2 h. All details of the primary and secondary antibodies used in this study are listed in Table S2. Signals were visualized using Immobilon™ Western Chemiluminescent HRP Substrate (Merck Millipore), captured using ImageQuant™ LAS 4000 Camera System (GE Healthcare, USA), and analyzed using ImageJ software (National Institutes of Health, USA). Actin and GAPDH served as internal controls. Means ± SE of normalized band intensities were denoted below corresponding bands in each picture.

### 2.5 Quantitative qRT-PCR

Total RNA was extracted from cells using TriPure Isolation Reagent (Roche), separated by 1-bromo-3-chloropropane, precipitated by isopropanol (J.T. Baker, USA), and finally reverse-transcribed into cDNA using MMLV Reverse Transcriptases (MMVL High Performance Reverse Transcriptase Kit; Lucigen, UK) and Oligo(dT)_20_ primers (Genomics, Taiwan). Gene expression was analyzed by Applied Biosystems™ TaqMan® Gene Expression assays (Thermo Fisher Scientific, USA). All TagMan® probes (FAM) used in this study are listed in Table S3. GAPDH served as an internal control. The relative concentration of target genes in the real-time PCR reaction (StepOnePlus™ Real-Time PCR Systems; Thermo Fisher Scientific) was determined by the threshold cycle (Ct) value, with differences between groups assessed using the 2-ΔΔCt method.

### 2.6 Plasmids and reporter assay

For exogenous expression, vectors carrying full-length EGFR (referred to as “EGFR”), full-length Akt with an N-terminal myristoylation signal tag (designated as “myr-Akt”, a constitutively active form of Akt), and full-length mTOR with an N-terminal Flag tag (labeled as “Flag-mTOR”) were all purchased from Addgene (USA). The C/EBP-β plasmid was kindly gifted by Dr. Kuo-Wei Chang. Empty vectors served as mock controls. For generating a promoter-driven reporter construct (Pp62) for p62/sqstm1 gene, restriction enzyme-based cloning methods were used. The promoter region of p62, ranging from −2126 to +438 [29], was amplified from human genomic DNA using PCR, cloned into a pGL3-Enhancer vector (Promega, USA), and joined with firefly luciferase-encoding sequences. The forward and reverse primers, containing *Bgl*Ⅱ and *Hin*dⅢ (New England Biolabs, USA) restriction sites respectively, were 5′-CCC AGA TCT GAT GAG GAA ATG AGA GGC TG-3′ and 5′-CCC AAG CTT CTT GGT CAC CAC TCC AGT CA-3’. The reporter construct was verified by restriction digestion and DNA sequencing, aligned to sequences based on publicly available data. For promoter activities determined by the dual-luciferase reporter system, the Pp62 plasmid (1.5 μg) was co-transfected with plasmids like EGFR (3 μg), myr-Akt (3 μg), and C/EBP-β (3 μg), plus the pRL-TK plasmid encoding renilla luciferase (20 ng; Promega), for 24 h. The firefly luciferase activities were detected using a luminometer (TECAN 200/200Pro; TECAN, Switzerland) and normalized against renilla luciferase activities.

### 2.7 Human subjects and tissue specimen collection

This study was approved by the Institutional Review Board of Taipei Veterans General Hospital (IRB-TPEVGH No.: 2016-02-005BCE, TVGH). Thirty-four pairs of tissue specimens were obtained from 58 OSCC patients who had undergone tumor resection surgery. Clinical parameters of these patients are described in Table S4. The specimens were fixed in 10% neutral buffered formalin, embedded in paraffin, and stored at the hospital's tissue bank until they were used.

### 2.8 Immunohistochemistry (IHC) staining

IHC was performed using the AutoProbe Ⅱ ABC universal staining kit (Bionexus, USA). Paraffin sections (4 μm thick) were dewaxed, rehydrated, and treated with 3% hydrogen peroxide. Antigen retrieval involved heating (≥95 °C) sections in pH 6.0 citrate buffer (10 mM; Genemed Biotechnologies, USA) using a pressure cooker. After blocking with 5% normal horse serum (Invitrogen, USA), sections were incubated overnight with the primary antibody. Controls excluded primary antibodies. Staining was visualized using AEC (3-amino-9-ethylcarbazole; Genemed Biotechnologies), and counterstained with hematoxylin (Muto Pure Chemicals, Japan). Staining densities were quantified from images of at least three random fields (200X) in each of the two specimens per patient and computed using pixel analysis, specifically by measuring the yellow (Y) value of the CMYK scale, using Photoshop software (Adobe, USA) [30].

### 2.9 Data analysis and statistics

Graphs were plotted using GraphPad Prism 9.0 software (USA). *t*-test, two-way ANOVA, log-rank test, and Fisher's exact test were used. Data are presented as the mean ± standard error of the mean (SEM). Differences between groups were considered significant at *p* < 0.05 (*); *p* < 0.01 (**); *p* < 0.001 (***); *p* < 0.0001 (****); “ns”, not significant.

## 3 Results

### 3.1 CDDP-induced p62 upregulation drives drug tolerance in OSCC

**Fig. 1:**
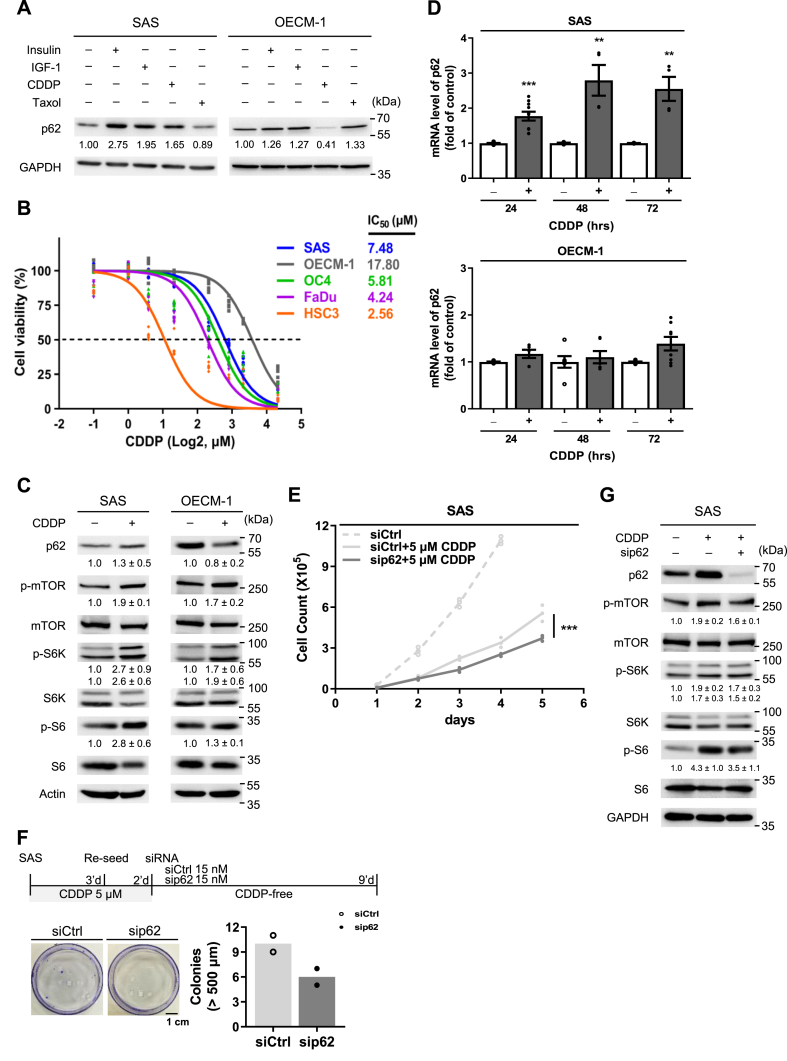
p62-mTOR modulates CDDP tolerance in SAS cells. (A) p62 levels in SAS and OECM-1 cells shown by Western blot, under the treatment of insulin, IGF-1, CDDP, or Taxol. Numbers represent average of normalized band intensities, based on at least duplicate analyses. (B) Dose-dependent cell viability curves of CDDP in five cancer cell lines (n = 6). (C) The effects of CDDP on p62 and mTOR pathway activation in SAS and OECM-1 cells. Numbers below immunoblotting bands represent means ± SE of normalized intensities (n = 3). (D) Time series of p62 mRNA levels upon CDDP treatment. Error bars indicate standard error of the mean (n = 3). P values were determined by two-tailed unpaired *t*-test with equal variance (**p < 0.01, ***p < 0.001). (E) Transient inhibition of p62 using sip62 and its effect on SAS cell survival. siCtrl, non-targeting siRNA control. P values were determined by two-way ANOVA test with equal variance (***p < 0.001; n = 3). (F) Effect of p62 inhibition on SAS colony formation upon CDDP treatment (n = 2). The top diagram depicts the experimental timeline. (G) p62 inhibition by sip62 abolishes the activating effect of CDDP on the p62-mTORC1 pathway. Numbers below each band represent means ± SE of normalized intensities (n = 3).

To explore p62's involvement in the acute CDDP response in OSCC, we set off by examining the p62-mTORC1 signaling in two OSCC cell lines, SAS and OECM-1, treated with CDDP (5 μM) and Taxol (20 nM) for 48 h. Insulin and insulin-like growth factor (IGF-1) were used to modulate mTORC1, leading to p62 accumulation (Fig. 1A) [31,32]. After CDDP treatment, SAS and OECM-1 showed increased and decreased p62 levels, respectively. Interestingly, Taxol treatment resulted in consistently opposite effects on p62 expression compared to CDDP in both cell lines (Fig. 1A). The variability in p62 expression between SAS and OECM-1(Fig. S1C) was reflected by CDDP tolerance, such that OECM-1 exhibited higher CDDP tolerance than SAS (Fig. 1B). Nonetheless, SAS and OECM-1 exhibited the highest CDDP tolerance among all of the cell lines tested, including OC4, FaDu, and HSC3 (Fig. 1B). Further investigation into the temporal dynamics showed that p62 mRNA levels consistently increased in SAS over a 72-h period, but remained constant in OECM-1 (Fig. 1D). Protein levels of p62 and downstream substrates of mTOR, such as p-S6K and p-S6, were upregulated in SAS but not OECM-1 upon CDDP treatment (Fig. 1C). These findings showed that SAS and OECM-1 present distinct patterns of p62-mTORC1 signaling in response to CDDP treatment, and that p62 levels could be decoupled from downstream events in the case of OECM-1.

To further examine whether p62 plays an active role in mediating CDDP susceptibility, we conducted a transient knockdown of p62 using sip62, in the CDDP-susceptible SAS cell line. p62 knockdown reduced both cell counts and colony formation, indicating p62 actively affects CDDP susceptibility in SAS (Fig. 1E and F). Our previous study showed that p62 knockdown alone does not inhibit growth [33]. Next, in the presence of CDDP, downregulation of p62 suppressed mTORC1 activation, as shown by lower levels of p-S6K and p-S6 (Fig. 1G). These results suggest that the p62-mTORC1 signaling pathway is not merely reactive, but actively contributes to CDDP tolerance in SAS cells.

### 3.2 p62 contributes to CDDP tolerance in drug-induced resistant cell subpopulations

**Fig. 2:**
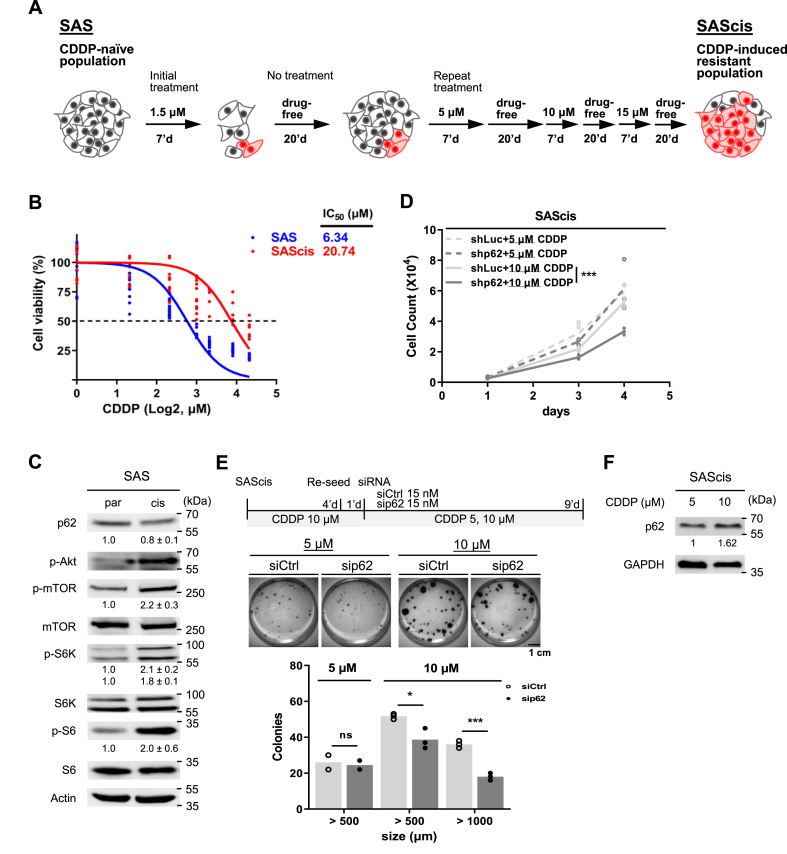
CDDP-induced drug resistance depends on p62-mTOR activity. (A) Schematics illustrating the process of inducing drug-resistant cells using CDDP. (B) Dose-dependent cell viability curves of SAS and SAScis (n = 6). (C) Downregulation of p62 and activation of the mTOR pathway in SAScis cells compared to SAS. Numbers below each band represent means ± SE of normalized intensities (n = 3). (D) Stable knockdown of p62 using shp62 and its effect on SAScis survival, in 5 or 10 μM of CDDP. shLuc, luciferase targeting shRNA control (n = 3). (E) Effect of p62 inhibition on SAScis colony formation in the presence of 5 or 10 μM of CDDP treatment. The top diagram depicts the experimental timeline. (F) CDDP induced p62 in SAScis cells. P values in (D, E) were determined using two-way ANOVA test or two-tailed unpaired *t*-test with equal variance (*p < 0.05, ***p < 0.001).

Given the varied influence of p62 in modulating drug responses in SAS and OECM-1, we extended to determine if p62 performs a similar function within drug-induced resistant states. For the generation of drug-induced resistant cell subpopulations, cells were sequentially exposed to progressively increased concentrations of CDDP. This exposure was periodically interrupted by drug-free periods (Fig. 2A), to facilitate the re-expansion of resistant cells within the original population. The emerging resistant cells, denoted as “SAScis”, displayed a markedly higher IC50 compared to the parental strain (Fig. 2B and Fig. S1A). Interestingly, SAScis demonstrated a signaling pattern characterized by enhanced mTORC1 activation (higher p-S6K and p-S6) and lowered p62 level (Fig. 2C), which is similar to the inherently resistant cell line, OECM-1 (Fig. 1A).

To determine whether the drug-induced resistant cell subpopulations maintained a dependency on p62 for their responsiveness to CDDP, we performed a stable knockdown of p62 using shp62 in SAScis cells (Fig. S1B). Following CDDP treatment, shp62 led to a significant reduction in both cell counts and colony formation, indicating that p62 contributes to the drug tolerance in drug-induced resistant cell subpopulations (Fig. 2D and E). Notably, the impact of p62 was observed only at a higher CDDP concentration of 10 μM, but not at the 5 μM concentration used for untrained parental cells (Fig. 1F). Furthermore, these subpopulations displayed dose-dependent p62 induction at 5 and 10 μM (Fig. 2F). These findings collectively underscore the ongoing pivotal role of p62 in drug-induced CDDP resistance, which coincides with the activation of the mTORC1 signaling complex.

### 3.3 p62 regulation through the EGFR-PI3K-Akt-C/EBP-β signaling axis

**Fig. 3:**
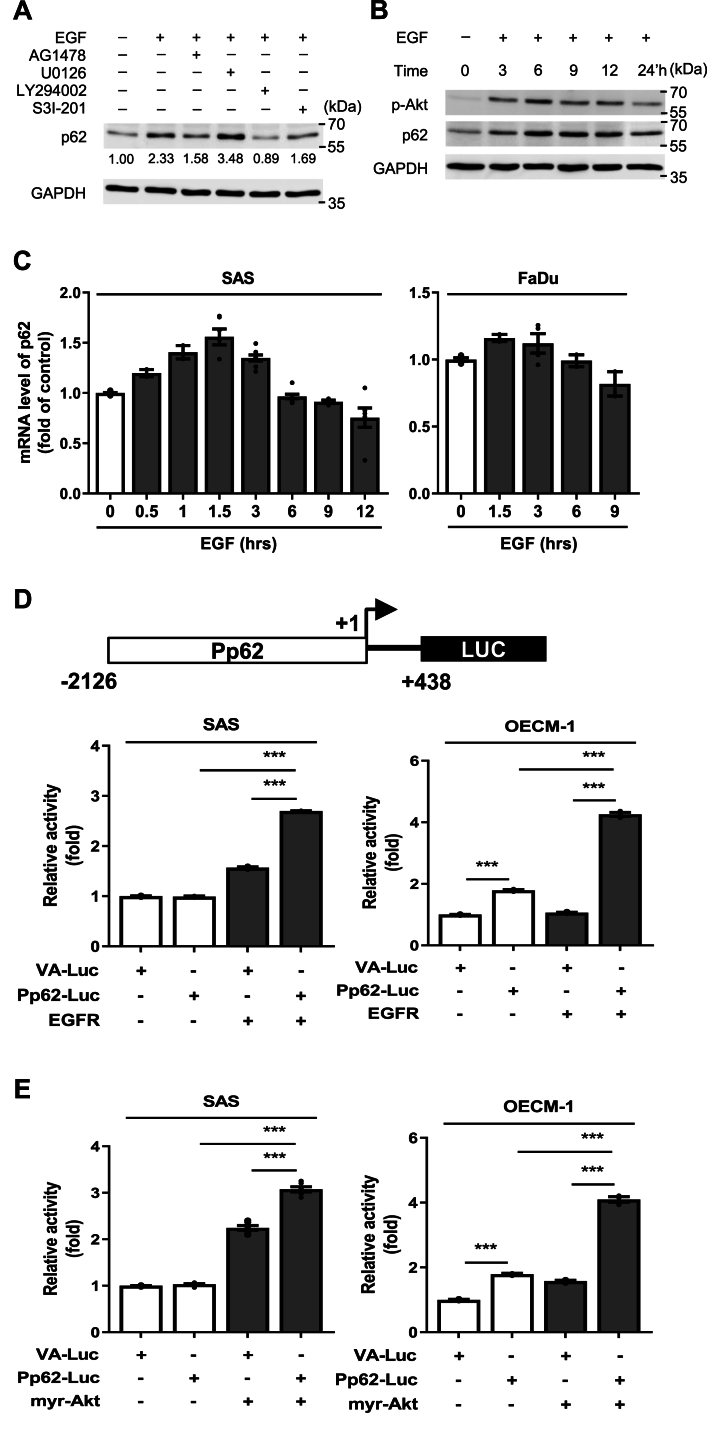
EGFR signaling induces p62 via the PI3K-Akt axis. (A) Effect of EGF and four EGFR-signaling inhibitors (AG1478, U0126, LY294002, and S3I-201) on p62 expression in SAS cells. Numbers represent average of normalized band intensities, based on at least duplicate analyses. Detailed schematics of the inhibitors and their corresponding molecular pathways was shown in Fig. 4F. (B) Time course of p62 and *p*-Akt levels following EGF induction. (C) Time series of p62 mRNA levels in SAS and FaDu cells induced by EGF. Error bars represent standard error of the mean (n = 3). (D, E) The top diagram depicts Pp62-Luc reporter construct. Both overexpressing EGFR and myr-Akt induced Pp62-Luc expression. VA-Luc, luciferase vector control. P values in were determined using two-tailed unpaired *t*-test with equal variance (***p < 0.001) (n = 3).

Next, we sought to elucidate the signaling pathway upstream of p62-mTORC1 during CDDP treatment. We hypothesized that the epidermal growth factor receptor (EGFR) is involved, given its critical role in the pathogenesis, drug resistance, and recurrence of HNSCC [18,34,35]. First, we used inhibitors to dissect the role of EGFR (AG1478) and three of its downstream molecules–PI3K/Akt (LY294002), MEK1/2 (U0126), and STAT3 (S3I-201)–on p62 expression in the absence of CDDP [[36], [37], [38]]. p62 levels were analyzed 12 h after treatment with epidermal growth factor (EGF). Western blot results showed that EGF-stimulated p62 increase could be attenuated by AG1478 and LY294002, but not U0126 and S3I-201 (Fig. 3A), indicating that EGF induced p62 via the PI3K-Akt route. Further, EGF stimulation experiments revealed that both *p*-Akt and p62 levels increased in a time-dependent manner, peaking at 6 h after EGF treatment (Fig. 3B and S2B). Such protein temporal dynamics coincided with p62 mRNA expression, which peaked at 1.5 h in both SAS and FaDu cells (Fig. 3C), demonstrating that EGFR-PI3K-Akt signaling is involved in transcriptionally upregulating p62. To further support the proposition of transcriptional mechanism, we constructed a reporter featuring the p62 promoter region, extending from −2126 upstream to +438 downstream of its coding region. Dual-luciferase assays demonstrated a significant increase in p62 promoter activity upon EGFR (Fig. 3D) and myr-Akt overexpression (Fig. 3E). Collectively, p62 upregulation is predominantly mediated through the EGFR-PI3K-Akt signaling pathway.

**Fig. 4:**
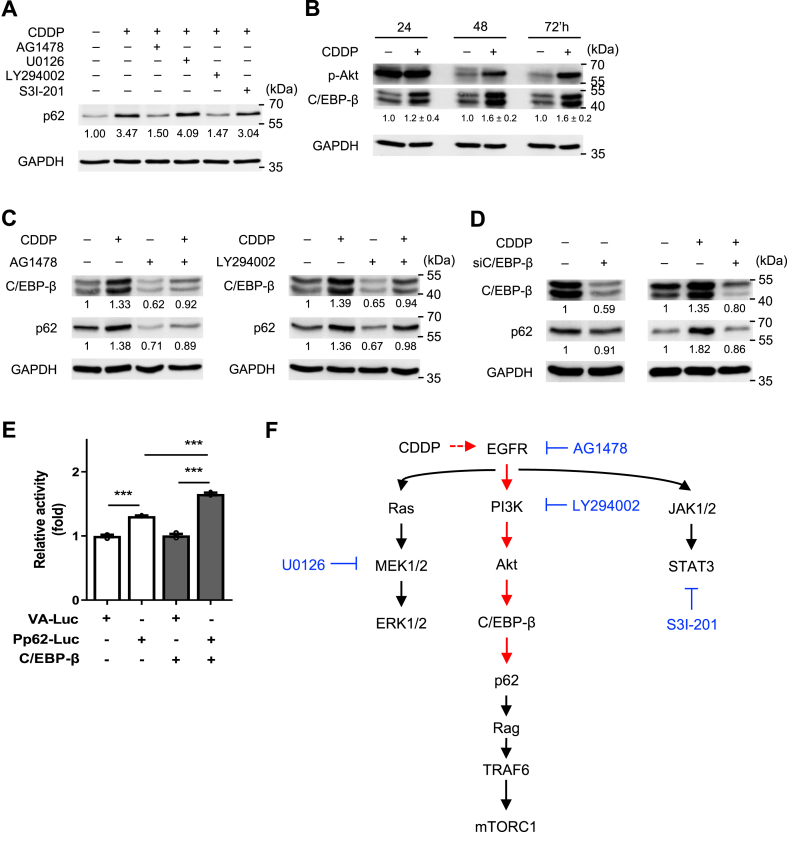
C/EBP-β mediates EGFR-PI3K-Akt signaling to p62 under CDDP treatment. (A) Effect of CDDP treatment and four EGFR-signaling inhibitors (AG1478, U0126, LY294002, and S3I-201) on p62 expression in SAS cells. (B) Time course of *p*-Akt and C/EBP-β levels following CDDP treatment. (C) Both AG1478 and LY294002 suppressed p62 and C/EBP-β upon CCDP treatment. (D) Inhibition of C/EBP-β using siC/EBP-β suppressed p62. (E) C/EBP-β overexpression induced Pp62-Luc reporter. VA-Luc, luciferase vector control. P values in were determined using two-tailed unpaired *t*-test with equal variance (***p < 0.001; n = 3). (F) Schematics showing relevant EGFR pathways and inhibitors (blue) used in this study. Red arrows indicate signaling effects demonstrated in this study. All numbers below the immunoblotting bands (A–D) represent average of normalized band intensities, based on at least duplicate analyses.

To further delve into the EGFR-PI3K-Akt-p62 regulatory pathway in response to CDDP treatment, we conducted the inhibitor screening assay in the presence of CDDP. The outcomes were similar to that observed with EGF stimulation, where AG1478 and LY294002 dampened the effect of CDDP (Fig. 4A). Next, we asked whether the transcription factor C/EBP-β (CCAAT enhancer binding protein β) could mediate the signaling downstream of Akt. We found that time-dependent elevations of C/EBP-β correlated with *p*-Akt after treatment with both EGF (Fig. S2) and CDDP (Fig. 4B). This led us to hypothesize a regulatory role for C/EBP-β, a predominant oncogenic transcription factor in breast cancer, modulated by several receptor tyrosine kinases, such as EGFR, fibroblast growth factor receptor (FGFR), insulin receptor (IR), and IGF-1 receptor (IGF-1R) [39,40]. Consistent with our prediction, Western blot analysis demonstrated that C/EBP-β was induced by CDDP, but suppressed by inhibitors for EGFR (AG1478) and PI3K/Akt (LY294002) (Fig. 4C), implying that C/EBP-β is involved in the EGFR-PI3K-Akt signaling axis. It is noted that p62 levels displayed a strong correlation with those of C/EBP-β when under the treatments of CDDP and EGFR/Akt inhibitors (Fig. 4C). In line with these findings, transient knockdown of C/EBP-β using siRNA resulted in diminished p62 levels during CDDP exposure (Fig. 4D). Concordantly, luciferase assay further confirmed enhancement of p62 promoter activity upon overexpression of C/EBP-β (Fig. 4E). Collectively, these findings establish that the EGFR-PI3K-Akt-C/EBP-β signaling axis orchestrates p62 upregulation during CDDP treatment (Fig. 4F).

### 3.4 High mTORC1 activity correlate with increased recurrence among patients with advanced OSCC

**Fig. 5:**
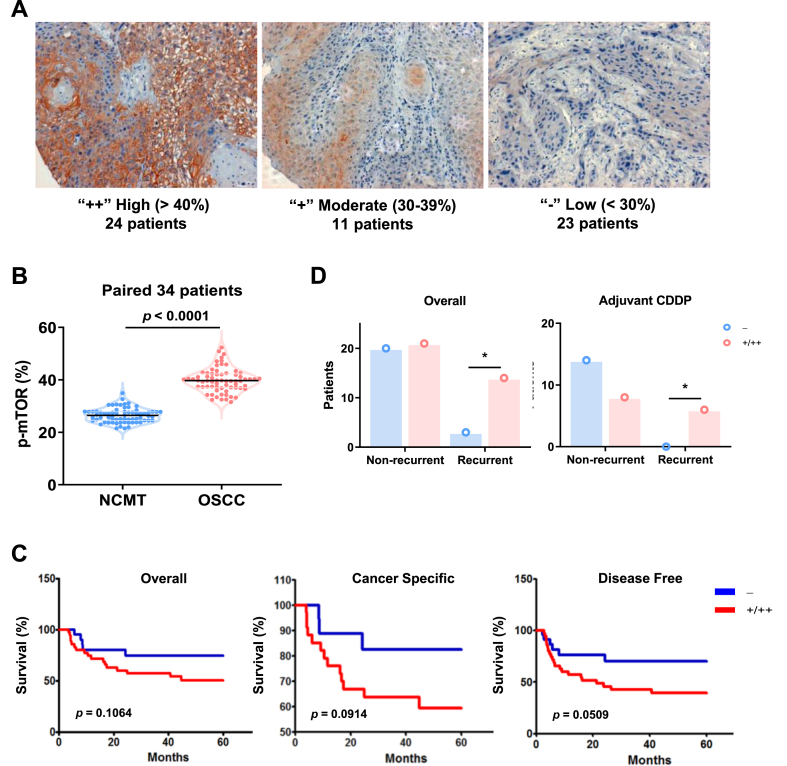
High mTOR activity correlates with poor prognosis in HNSCC patients. (A) Immunohistochemistry of OSCC tumor specimens displaying *p*-mTOR expression. Three representative images for high, moderate, and low levels of *p*-mTOR were shown. (B) Significantly higher levels of *p*-mTOR in OSCC tumor specimens relative to paired NCMT tissues among 34 patients. Statistical analysis was based on two-tailed paired *t*-test. (C) Patient survival curves comparing moderate/high (+/++) to low (−) expression levels of *p*-mTOR. P values were determined by log-rank test. (D) Recurrence of OSCC was associated with *p*-mTOR expression. P values were determined by Fisher's exact test.

Given the pronounced activity of p62-mTORC1 observed in both innate and drug-induced CDDP resistance during cellular assays, we aim to further investigate the potential role of mTORC1 in real-world clinical patients. We collected tumor specimens from 58 OSCC patients, of which 34 patients provided paired tumor and non-cancerous matched tissue (NCMT) samples. 28 patients exhibited advanced diseases and underwent postoperative adjuvant CDDP-based combinational treatments. Given the fact that the phosphorylation level at mTOR S2448 is indicative of mTORC1 activity [41], we utilized immunohistochemical staining with p-S2448 mTOR antibody. This allowed us to classify the tumors by mTORC1 activity into “low”, “moderate”, and “high” groups, based on the fraction of mTORC1-positive cells (Fig. 5A). The staining activity was categorized by two pathologists independently, then aggregated by consensus. Our results showed significantly elevated *p*-mTOR levels in tumor tissues compared to NCMT (*p* < 0.0001) (Fig. 5B), underscoring mTORC1's dominant role in OSCC. For survival analysis, we merged the “moderate” and “high” groups and compared them with the “low” group on various clinical parameters. No significant differences were observed in tumor size, lymph node metastasis, or differentiation status between the two groups (Fig. S3). While Kaplan-Meier survival analysis revealed no marked differences in overall and cancer-specific survival between the two groups, a noticeably improved disease-free survival (DFS) was evident in the low group (Fig. 5C). However, in the subset of patients with advanced disease who received postoperative adjuvant CDDP treatment, those with elevated mTORC1 activity had a significantly higher tumor recurrence rate (Fig. 5D). Together, these immunohistochemical results demonstrated that the activity mTORC1 is predictive of OSCC prognosis and treatment response.

### 3.5 mTORC1 activity is associated with cancer stem cell markers, suggesting therapeutic potential

**Fig. 6:**
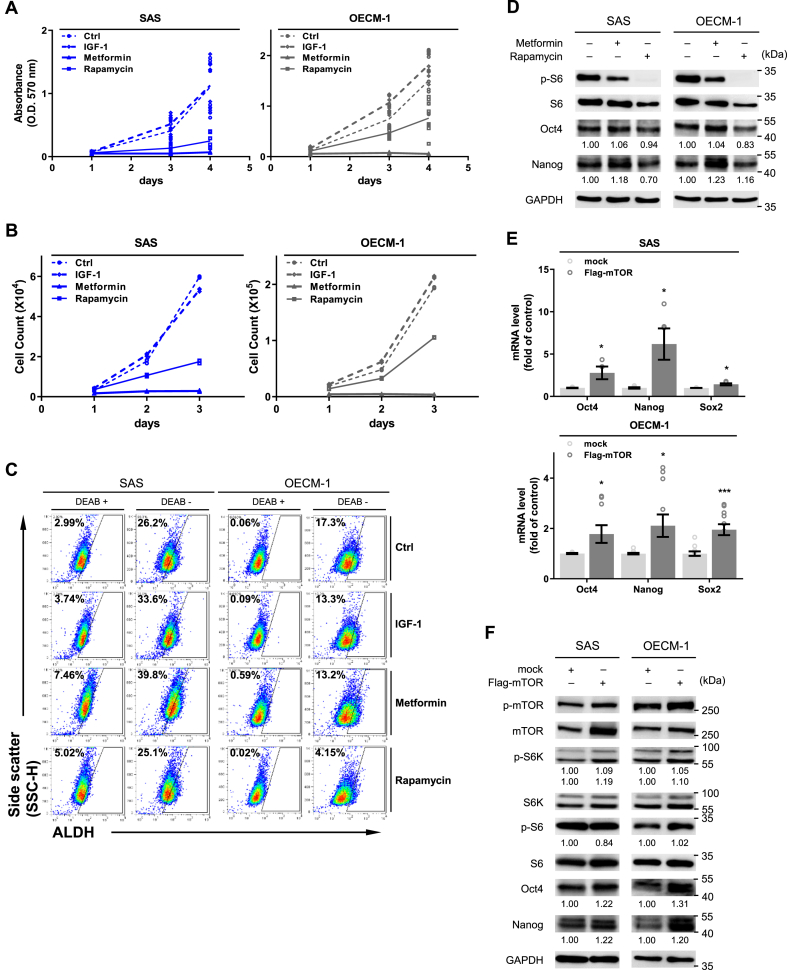
mTOR activity influences stemness in OSCC cells. (A, B) Influence of mTOR inhibition using Rapamycin and Metformin on the proliferative capacity of SAS and OECM-1 cells: (A) MTT assay (n = 6); (B) trypan-blue exclusion assay (n = 3). (C) ALDH^+^ cell populations quantified post-mTOR inhibition, using Aldefluor assay and flow cytometry. DEAB was used as an ALDH-specific inhibitor for negative control. IGF-1 was used as a positive control in (A–C). (D) Western blot analysis showing Oct4 and Nanog expression after mTOR inhibition. (E, F) Effects of mTOR overexpression on stemness markers (Oct4, Nanog, and Sox2) at mRNA (E) and protein (F) levels. Error bars indicate standard error of the mean (n = 3). P values were determined by two-tailed unpaired *t*-test with equal variance (*p < 0.05, ***p < 0.001). Band intensities in (D, F) were normalized and then averaged, based on at least duplicate analyses.

**Fig. 7:**
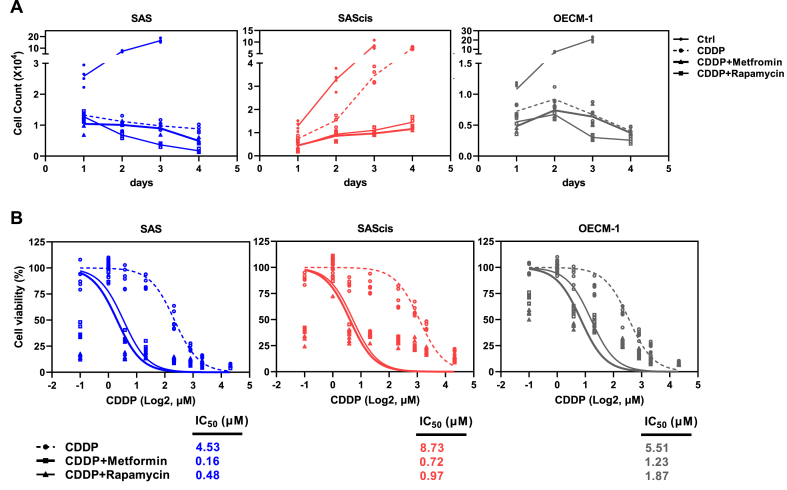
Inhibition of mTOR activity sensitizes SAS, SAScis, and OECM-1 cells to CDDP. Combinatorial treatment of Rapamycin or Metformin with CDDP on the viability of SAS, SAScis, and OECM-1 cells: (A) trypan-blue exclusion assay (n = 4); (B) MTT assay (n = 6).

Given the role of mTORC1 in drug resistance and patient survival, we then explored the potential therapeutic effect of two mTOR-inhibiting drugs, Rapamycin and Metformin, using OSCC cell lines. The two drugs were chosen due to their distinct targets: Rapamycin directly targets mTOR, whereas Metformin targets AMPK, an upstream signaling molecule that activates mTOR [42,43]. A previous study on triple-negative breast cancer showed that mTORC1/2 inhibitors enrich cancer stem cell (CSC) populations, which constitute a subpopulation of drug-resistant cells with self-renewing and tumor initiating capabilities [44]. For cell survival, we observed that both Rapamycin and Metformin resulted in significant reductions in cell proliferation (Fig. 6A and B). More importantly, the inhibitory effects of both Rapamycin and Metformin were observed in the presence of CDDP on cell proliferation and viability (Fig. 7). Among the three cell lines, SAScis — the CDDP-resistant strain — was the most strongly inhibited by Rapamycin and Metformin treatment, resulting in a 10-fold reduction in the IC50 of CDDP (middle panel of Fig. 7A and B).

To explore the potential mechanism by which Rapamycin and Metformin inhibits cell survival, we used flow cytometry to examine the activity of aldehyde dehydrogenase (ALDH), a cancer stem cell marker. Rapamycin significantly decreased ALDH activity in both SAS and OECM-1 cells. Intriguingly, Metformin increased ALDH activity to levels comparable to those seen with IGF-1 stimulation (Fig. 6C). The varied effects of Rapamycin and Metformin on ALDH were consistent with their effects on two other stem cell markers, Oct4 and Nanog. Nonetheless, both agents effectively suppressed mTORC1, as indicated by lowered p-S6 level (Fig. 6D). Lastly, to establish the link between mTORC1 activity and stemness markers, we demonstrated that ectopic overexpression of flag-mTOR in both SAS and OECM-1 cells significantly elevated both mRNA (Fig. 6E) and protein (Fig. 6F) levels of Oct4, Nanog, and Sox2. These findings collectively indicate the association between mTORC1 and cancer stemness. However, variability in therapeutic outcomes and the underlying mechanism still require further investigation.

## 4 Discussion

Systemic administration of a cisplatin-based combination regimen offers notable survival benefits to OSCC patients. Hence, overcoming both innate and acquired resistance to cisplatin remains a critical challenge in preventing therapeutic failure. In this study, we showed that CDDP-induced p62 upregulation, facilitated by the EGFR-PI3K-Akt-C/EBP-β signaling axis, activates mTORC1 complex, thereby enhancing drug tolerance in OSCC. Elevated mTORC1 activity correlates with both higher recurrence in advanced patients and cancer stem cell characteristics, underscoring its therapeutic potential.

The cellular and molecular landscapes of cancer markedly differ from those of normal tissues. In normal cells, mTORC1 activation is governed by various factors (e.g. ATP and amino acids) and stimuli like insulin and IGF-1, which bind to receptor tyrosine kinases (RTKs) and G protein-coupled receptors (GPCRs) [31]. On the contrary, cancer cells require sustained and elevated mTORC1 activity to bolster proliferation and to evade cytotoxic effects of chemo- and targeted therapy. This aberrant activity is driven by various oncogenic pathways and dysfunctions in tumor suppressors, including mutated or amplified EGFR, PI3K, Akt, Ras, and Raf, as well as impaired PTEN, APC, LKB1/STK11, and Gator1 [45]. In lung cancer, tumors with acquired chemoresistance showed constitutive activation of mTOR signaling and autophagy defects, with high steady-state levels of LC3 and p62 [46,47]. Conversely, in liver cancer, CDDP-induced drug resistance correlates with lowered mTOR signaling with increased autophagy, as evidenced by elevated Beclin-1, increased LC3B-II, and reduced p62 [48,49]. Similar findings have emerged in studies of testicular cancer [50], ovarian cancer [[51], [52], [53]], and cervical cancer [54], in which autophagy inhibition confers CDDP sensitivity through mTOR activation. In head and neck cancer, the PI3K-Akt-mTORC1 signaling becomes deregulated and activated in CDDP-induced resistant cells [55]. Furthermore, the regulatory-associated protein of mTOR (Raptor), a crucial scaffold protein in the mTORC1 signaling pathway, serves as an independent predictor of both DFS and OS; in contrast, mTOR and its downstream molecules, S6K and S6, show no association with outcomes in HNSCC patients. These findings are consistent with our work that elevated *p*-mTOR (phosphorylated at S2448) [41] correlates with a higher recurrence rate in advanced OSCC patients.

Although mTORC1 signaling and autophagy consistently emerge as common themes across various cancer types, specific nuances differ, possibly reflecting differences in cell lineages. In our work, such nuanced differences were observed between SAS and OECM-1, in which p62 expressions exhibited opposite trends and may be decoupled from downstream events in the case of OECM-1. Interestingly, autophagy may be irrelevant in OSCC cells with acquired CDDP resistance, as only CDDP-sensitive SCC-4 cells displayed a notable LC3B–I to LC3B-II conversion upon CDDP treatment, a change not observed in SCC-4cisR cells [56]. The inconsistent effect of mTORC1 was also exemplified in CaSki cells, which present higher CDDP sensitivity and p62 levels compared to HeLa cells [54]. A similar pattern was observed in cervical cancer: patients responsive to CDDP consistently display higher p62 levels than non-responders [54]. Notably, in OSCC, we found a correlation between elevated p62 and CDDP sensitivity. Moreover, despite reduced p62 levels in CDDP-induced resistant cells, it continued to play an active role in weakening drug sensitivity through mTORC1 activation.

In addition to the pivotal role of mTORC1 in cancers, numerous studies have shown that p62 is abundantly expressed in various cancers, fostering tumor progression through modulating cell proliferation, inflammation, apoptosis, and autophagy [57]. p62 is transcriptionally regulated by the Ras-MEK-ERK1/2 [58], Keap1-Nrf2 [29,59], and JNK/c-Jun pathways [60], and also through post-translational autophagic degradation. Regarding downstream events, p62 serves not only as a reporter for autophagy, but also facilitates proteasomal degradation of ubiquitinated proteins (e.g. tau), eventually impacting cell survival [61]. Our work demonstrated the role of p62 in strengthening mTORC1 activation via the EGFR-PI3K-Akt-C/EBP-β signaling cascade, promoting chemoresistance of OSCC. Finally, given the variability of p62 and mTOR's role in different types of cancers, and their complex interplay with various growth factor signals, further investigation tailored toward specific treatment circumstances is required for mTOR-based therapy.

## 5 Conclusions

Our study demonstrates the regulatory axis in which EGFR-PI3K-Akt-C/EBP-β pathway upregulates p62, thereby initiating the mTORC1 signaling cascade to promote CDDP resistance in OSCC. Moreover, our findings provide clinical implications by showing the potential of targeting mTORC1 as a therapeutic strategy in managing OSCC. Given the diverse roles of p62 and mTOR across different cancer contexts and treatment scenarios, further investigations into their prognostic utilities and therapeutic potential is advisable.

## CRediT authorship contribution statement

**Hsiu-Chuan Chang:** Writing – original draft, Validation, Software, Methodology, Investigation, Formal analysis, Data curation, Conceptualization. **Cheng-Chieh Yang:** Validation, Supervision, Resources, Project administration, Investigation, Funding acquisition, Conceptualization. **Lai-Keng Loi:** Software, Formal analysis, Data curation. **Chi-Hsun Hung:** Software, Formal analysis, Data curation. **Cheng-Hsien Wu:** Resources, Data curation, Conceptualization. **Yu-Cheng Lin:** Writing – review & editing, Writing – original draft, Validation, Supervision, Project administration, Funding acquisition.

## Declaration of generative AI and AI-assisted technologies in the writing process

During the preparation of this work the authors used ChatGPT (GPT-4 September 25, 2023) for grammatical correction and semantic recommendation. After using ChatGPT, the authors reviewed and edited the content as needed and take full responsibility for the content of the publication.

## Declaration of competing interest

The authors declare that they have no known competing financial interests or personal relationships that could have appeared to influence the work reported in this paper.

## References

[1] Siegel R.L., Miller K.D., Fuchs H.E., Jemal A. (2021). Cancer statistics, 2021. CA A Cancer J. Clin..

[2] Siegel R.L., Miller K.D., Fuchs H.E., Jemal A. (2022). Cancer statistics, 2022. CA A Cancer J. Clin..

[3] Tsai Y.-T., Chen W.-C., Hsu C.-M., Tsai M.-S., Chang G.-H., Lee Y.-C., Huang E.I., Fang C.-C., Lai C.-H. (2021). Survival-weighted Health profiles in patients treated for advanced oral cavity squamous cell carcinoma. Front. Oncol..

[4] Lo W.-L., Kao S.-Y., Chi L.-Y., Wong Y.-K., Chang R.C.-S. (2003). Outcomes of oral squamous cell carcinoma in Taiwan after surgical therapy: factors affecting survival. J. Oral Maxillofac. Surg..

[5] Wang B., Zhang S., Yue K., Wang X.-D. (2013). The recurrence and survival of oral squamous cell carcinoma: a report of 275 cases. Chin. J. Cancer.

[6] Florea A.M., Büsselberg D. (2011). Cisplatin as an anti-tumor drug: cellular mechanisms of activity, drug resistance and induced side effects. Cancers.

[7] Dasari S., Tchounwou P.B. (2014). Cisplatin in cancer therapy: molecular mechanisms of action. Eur. J. Pharmacol..

[8] Galluzzi L., Senovilla L., Vitale I., Michels J., Martins I., Kepp O., Castedo M., Kroemer G. (2012). Molecular mechanisms of cisplatin resistance. Oncogene.

[9] Redmond K.M., Wilson T.R., Johnston P.G., Longley D.B. (2008). Resistance mechanisms to cancer chemotherapy. Front. Biosci..

[10] Recasens A., Munoz L. (2019). Targeting cancer cell dormancy. Trends Pharmacol. Sci..

[11] Trumpp A., Wiestler O.D. (2008). Mechanisms of Disease: cancer stem cells--targeting the evil twin. Nat. Clin. Pract. Oncol..

[12] Glasspool R.M., Teodoridis J.M., Brown R. (2006). Epigenetics as a mechanism driving polygenic clinical drug resistance. Br. J. Cancer.

[13] Sharma S.V., Lee D.Y., Li B., Quinlan M.P., Takahashi F., Maheswaran S., McDermott U., Azizian N., Zou L., Fischbach M.A., Wong K.-K., Brandstetter K., Wittner B., Ramaswamy S., Classon M., Settleman J. (2010). A chromatin-mediated reversible drug-tolerant state in cancer cell subpopulations. Cell.

[14] Guler G.D., Tindell C.A., Pitti R., Wilson C., Nichols K., KaiWai Cheung T., Kim H.-J., Wongchenko M., Yan Y., Haley B., Cuellar T., Webster J., Alag N., Hegde G., Jackson E., Nance T.L., Giresi P.G., Chen K.-B., Liu J., Jhunjhunwala S., Settleman J., Stephan J.-P., Arnott D., Classon M. (2017). Repression of stress-induced LINE-1 expression protects cancer cell subpopulations from lethal drug exposure. Cancer Cell.

[15] Rehman S.K., Haynes J., Collignon E., Brown K.R., Wang Y., Nixon A.M.L., Bruce J.P., Wintersinger J.A., Singh Mer A., Lo E.B.L., Leung C., Lima-Fernandes E., Pedley N.M., Soares F., McGibbon S., He H.H., Pollet A., Pugh T.J., Haibe-Kains B., Morris Q., Ramalho-Santos M., Goyal S., Moffat J., O'Brien C.A. (2021). Colorectal cancer cells enter a diapause-like DTP state to survive chemotherapy. Cell.

[16] Wee P., Wang Z. (2017). Epidermal growth factor receptor cell proliferation signaling pathways. Cancers.

[17] Li A., Cao W., Liu X., Zhang Y., Ma Y., Xu R., Zhang R., Liu X., Zhou S., Wang R., Liu J., Tang X. (2020). Gefitinib sensitization of cisplatin-resistant wild-type EGFR non-small cell lung cancer cells. J. Cancer Res. Clin. Oncol..

[18] Oh S.J., Lim J.Y., Son M.K., Ahn J.H., Song K.-H., Lee H.-J., Kim S., Cho E.H., Chung J.-Y., Cho H., Kim H., Kim J.-H., Park J., Choi J., Hwang S.W., Kim T.W. (2023). TRPV1 inhibition overcomes cisplatin resistance by blocking autophagy-mediated hyperactivation of EGFR signaling pathway. Nat. Commun..

[19] Hiraishi Y., Wada T., Nakatani K., Tojyo I., Matsumoto T., Kiga N., Negoro K., Fujita S. (2008). EGFR inhibitor enhances cisplatin sensitivity of oral squamous cell carcinoma cell lines. Pathol. Oncol. Res..

[20] Zou Z., Tao T., Li H., Zhu X. (2020). mTOR signaling pathway and mTOR inhibitors in cancer: progress and challenges. Cell Biosci..

[21] Corcoran R.B., Rothenberg S.M., Hata A.N., Faber A.C., Piris A., Nazarian R.M., Brown R.D., Godfrey J.T., Winokur D., Walsh J., Mino-Kenudson M., Maheswaran S., Settleman J., Wargo J.A., Flaherty K.T., Haber D.A., Engelman J.A. (2013). TORC1 suppression predicts responsiveness to RAF and MEK inhibition in BRAF-mutant melanoma. Sci. Transl. Med..

[22] Elkabets M., Vora S., Juric D., Morse N., Mino-Kenudson M., Muranen T., Tao J., Campos A.B., Rodon J., Ibrahim Y.H., Serra V., Rodrik-Outmezguine V., Hazra S., Singh S., Kim P., Quadt C., Liu M., Huang A., Rosen N., Engelman J.A., Scaltriti M., Baselga J. (2013). mTORC1 inhibition is required for sensitivity to PI3K p110α inhibitors in PIK3CA-mutant breast cancer. Sci. Transl. Med..

[23] Kawabata S., Mercado-Matos J.R., Hollander M.C., Donahue D., Wilson W., Regales L., Butaney M., Pao W., Wong K.-K., Jänne P.A., Dennis P.A. (2014). Rapamycin prevents the development and progression of mutant epidermal growth factor receptor lung tumors with the acquired resistance mutation T790M. Cell Rep..

[24] Wang Z., Martin D., Molinolo A.A., Patel V., Iglesias-Bartolome R., Degese M.S., Vitale-Cross L., Chen Q., Gutkind J.S. (2014). mTOR co-targeting in cetuximab resistance in head and neck cancers harboring PIK3CA and RAS mutations. J. Natl. Cancer Inst..

[25] Duran A., Amanchy R., Linares J.F., Joshi J., Abu-Baker S., Porollo A., Hansen M., Moscat J., Diaz-Meco M.T. (2011). p62 is a key regulator of nutrient sensing in the mTORC1 pathway. Mol. Cell..

[26] Matherne M.G., Phillips E.S., Embrey S.J., Burke C.M., Machado H.L. (2023). Emerging functions of C/EBPβ in breast cancer. Front. Oncol..

[27] Phi L.T.H., Sari I.N., Yang Y.-G., Lee S.-H., Jun N., Kim K.S., Lee Y.K., Kwon H.Y. (2018). Cancer stem cells (CSCs) in drug resistance and their therapeutic implications in cancer treatment. Stem Cell. Int..

[28] Loi L.-K., Yang C.-C., Lin Y.-C., Su Y.-F., Juan Y.-C., Chen Y.-H., Chang H.-C. (2023). Decoy peptides effectively inhibit the binding of SARS-CoV-2 to ACE2 on oral epithelial cells. Heliyon.

[29] Jain A., Lamark T., Sjøttem E., Larsen K.B., Awuh J.A., Øvervatn A., McMahon M., Hayes J.D., Johansen T. (2010). p62/SQSTM1 is a target gene for transcription factor NRF2 and creates a positive feedback loop by inducing antioxidant response element-driven gene transcription. J. Biol. Chem..

[30] Pham N.-A., Morrison A., Schwock J., Aviel-Ronen S., Iakovlev V., Tsao M.-S., Ho J., Hedley D.W. (2007). Quantitative image analysis of immunohistochemical stains using a CMYK color model. Diagn. Pathol..

[31] Dancey J. (2010). mTOR signaling and drug development in cancer. Nat. Rev. Clin. Oncol..

[32] Hua F., Li K., Yu J.-J., Lv X.-X., Yan J., Zhang X.-W., Sun W., Lin H., Shang S., Wang F., Cui B., Mu R., Huang B., Jiang J.-D., Hu Z.-W. (2015). TRB3 links insulin/IGF to tumour promotion by interacting with p62 and impeding autophagic/proteasomal degradations. Nat. Commun..

[33] Yeh L.-Y., Liu C.-J., Wong Y.-K., Chang C., Lin S.-C., Chang K.-W. (2015). miR-372 inhibits p62 in head and neck squamous cell carcinoma in vitro and in vivo. Oncotarget.

[34] Ang K.K., Berkey B.A., Tu X., Zhang H.-Z., Katz R., Hammond E.H., Fu K.K., Milas L. (2002). Impact of epidermal growth factor receptor expression on survival and pattern of relapse in patients with advanced head and neck carcinoma. Cancer Res..

[35] Wheeler D.L., Dunn E.F., Harari P.M. (2010). Understanding resistance to EGFR inhibitors-impact on future treatment strategies. Nat. Rev. Clin. Oncol..

[36] Han Y., Caday C.G., Nanda A., Cavenee W.K., Huang H.J. (1996). Tyrphostin AG 1478 preferentially inhibits human glioma cells expressing truncated rather than wild-type epidermal growth factor receptors. Cancer Res..

[37] Siddiquee K., Zhang S., Guida W.C., Blaskovich M.A., Greedy B., Lawrence H.R., Yip M.L.R., Jove R., McLaughlin M.M., Lawrence N.J., Sebti S.M., Turkson J. (2007). Selective chemical probe inhibitor of Stat3, identified through structure-based virtual screening, induces antitumor activity. Proc. Natl. Acad. Sci. U.S.A..

[38] Li Q., Tie Y., Alu A., Ma X., Shi H. (2023). Targeted therapy for head and neck cancer: signaling pathways and clinical studies. Signal Transduct. Targeted Ther..

[39] Zahnow C.A. (2009). CCAAT/enhancer-binding protein beta: its role in breast cancer and associations with receptor tyrosine kinases. Expet Rev. Mol. Med..

[40] Lu W.-C., Kao S.-Y., Yang C.-C., Tu H.-F., Wu C.-H., Chang K.-W., Lin S.-C. (2014). EGF up-regulates miR-31 through the C/EBPβ signal cascade in oral carcinoma. PLoS One.

[41] Rosner M., Siegel N., Valli A., Fuchs C., Hengstschläger M. (2010). mTOR phosphorylated at S2448 binds to raptor and rictor. Amino Acids.

[42] Umar A., Dunn B.K., Greenwald P. (2012). Future directions in cancer prevention. Nat. Rev. Cancer.

[43] Occhiuzzi M.A., Lico G., Ioele G., De Luca M., Garofalo A., Grande F. (2023). Recent advances in PI3K/PKB/mTOR inhibitors as new anticancer agents. Eur. J. Med. Chem..

[44] Bhola N.E., Jansen V.M., Koch J.P., Li H., Formisano L., Williams J.A., Grandis J.R., Arteaga C.L. (2016). Treatment of triple-negative breast cancer with TORC1/2 inhibitors sustains a drug-resistant and notch-dependent cancer stem cell population. Cancer Res..

[45] Ilagan E., Manning B.D. (2016). Emerging role of mTOR in the response to cancer therapeutics. Trends Cancer Res..

[46] Gremke N., Polo P., Dort A., Schneikert J., Elmshäuser S., Brehm C., Klingmüller U., Schmitt A., Reinhardt H.C., Timofeev O., Wanzel M., Stiewe T. (2020). mTOR-mediated cancer drug resistance suppresses autophagy and generates a druggable metabolic vulnerability. Nat. Commun..

[47] Shen C., Shyu D.-L., Xu M., Yang L., Webb A., Duan W., Williams T.M. (2022). Deregulation of AKT-mTOR signaling contributes to chemoradiation resistance in lung squamous cell carcinoma. Mol. Cancer Res..

[48] Singh M.P., Cho H.J., Kim J.-T., Baek K.E., Lee H.G., Kang S.C. (2019). Morin hydrate reverses cisplatin resistance by impairing PARP1/HMGB1-dependent autophagy in hepatocellular carcinoma. Cancers.

[49] Luo L., Sun W., Zhu W., Li S., Zhang W., Xu X., Fang D., Grahn T.H.M., Jiang L., Zheng Y. (2021). BCAT1 decreases the sensitivity of cancer cells to cisplatin by regulating mTOR-mediated autophagy via branched-chain amino acid metabolism. Cell Death Dis..

[50] Yuan M., Yao Y., Wu D., Zhu C., Dong S., Tong X. (2022). Pannexin1 inhibits autophagy of cisplatin-resistant testicular cancer cells by mediating ATP release. Cell Cycle.

[51] Ma H., Li Y., Wang X., Wu H., Qi G., Li R., Yang N., Gao M., Yan S., Yuan C., Kong B. (2019). PBK, targeted by EVI1, promotes metastasis and confers cisplatin resistance through inducing autophagy in high-grade serous ovarian carcinoma. Cell Death Dis..

[52] Qi G., Ma H., Li Y., Peng J., Chen J., Kong B. (2021). TTK inhibition increases cisplatin sensitivity in high-grade serous ovarian carcinoma through the mTOR/autophagy pathway. Cell Death Dis..

[53] Liu L., Sun Y.-H., An R., Cheng R.-J., Li N., Zheng J.-H. (2023). LDLR promotes autophagy-mediated cisplatin resistance in ovarian cancer associated with the PI3K/AKT/mTOR signaling pathway. Kaohsiung J. Med. Sci..

[54] Huang H., Han Q., Zheng H., Liu M., Shi S., Zhang T., Yang X., Li Z., Xu Q., Guo H., Lu F., Wang J. (2021). MAP4K4 mediates the SOX6-induced autophagy and reduces the chemosensitivity of cervical cancer. Cell Death Dis..

[55] Niehr F., Eder T., Pilz T., Konschak R., Treue D., Klauschen F., Bockmayr M., Türkmen S., Jöhrens K., Budach V., Tinhofer I. (2018). Multilayered omics-based analysis of a head and neck cancer model of cisplatin resistance reveals intratumoral heterogeneity and treatment-induced clonal selection. Clin. Cancer Res..

[56] Magnano S., Hannon Barroeta P., Duffy R., O'Sullivan J., Zisterer D.M. (2021). Cisplatin induces autophagy-associated apoptosis in human oral squamous cell carcinoma (OSCC) mediated in part through reactive oxygen species. Toxicol. Appl. Pharmacol..

[57] Zhang X., Dai M., Li S., Li M., Cheng B., Ma T., Zhou Z. (2023). The emerging potential role of p62 in cancer treatment by regulating metabolism. Trends Endocrinol. Metabol..

[58] Duran A., Linares J.F., Galvez A.S., Wikenheiser K., Flores J.M., Diaz-Meco M.T., Moscat J. (2008). The signaling adaptor p62 is an important NF-kappaB mediator in tumorigenesis. Cancer Cell.

[59] Komatsu M., Kurokawa H., Waguri S., Taguchi K., Kobayashi A., Ichimura Y., Sou Y.-S., Ueno I., Sakamoto A., Tong K.I., Kim M., Nishito Y., Iemura S.-I., Natsume T., Ueno T., Kominami E., Motohashi H., Tanaka K., Yamamoto M. (2010). The selective autophagy substrate p62 activates the stress responsive transcription factor Nrf2 through inactivation of Keap1. Nat. Cell Biol..

[60] Puissant A., Fenouille N., Auberger P. (2012). When autophagy meets cancer through p62/SQSTM1. Am. J. Cancer Res..

[61] Liu W.J., Ye L., Huang W.F., Guo L.J., Xu Z.G., Wu H.L., Yang C., Liu H.F. (2016). p62 links the autophagy pathway and the ubiqutin-proteasome system upon ubiquitinated protein degradation. Cell. Mol. Biol. Lett..

