# Supplementary figures and images for: Interplay of p62-mTORC1 and EGFR signaling promotes cisplatin resistance in oral cancer
Source: Heliyon. 2024 Mar 21;10(6):e28406. doi: 10.1016/j.heliyon.2024.e28406 (PMC10979205; doi:10.1016/j.heliyon.2024.e28406)

## Slide 1
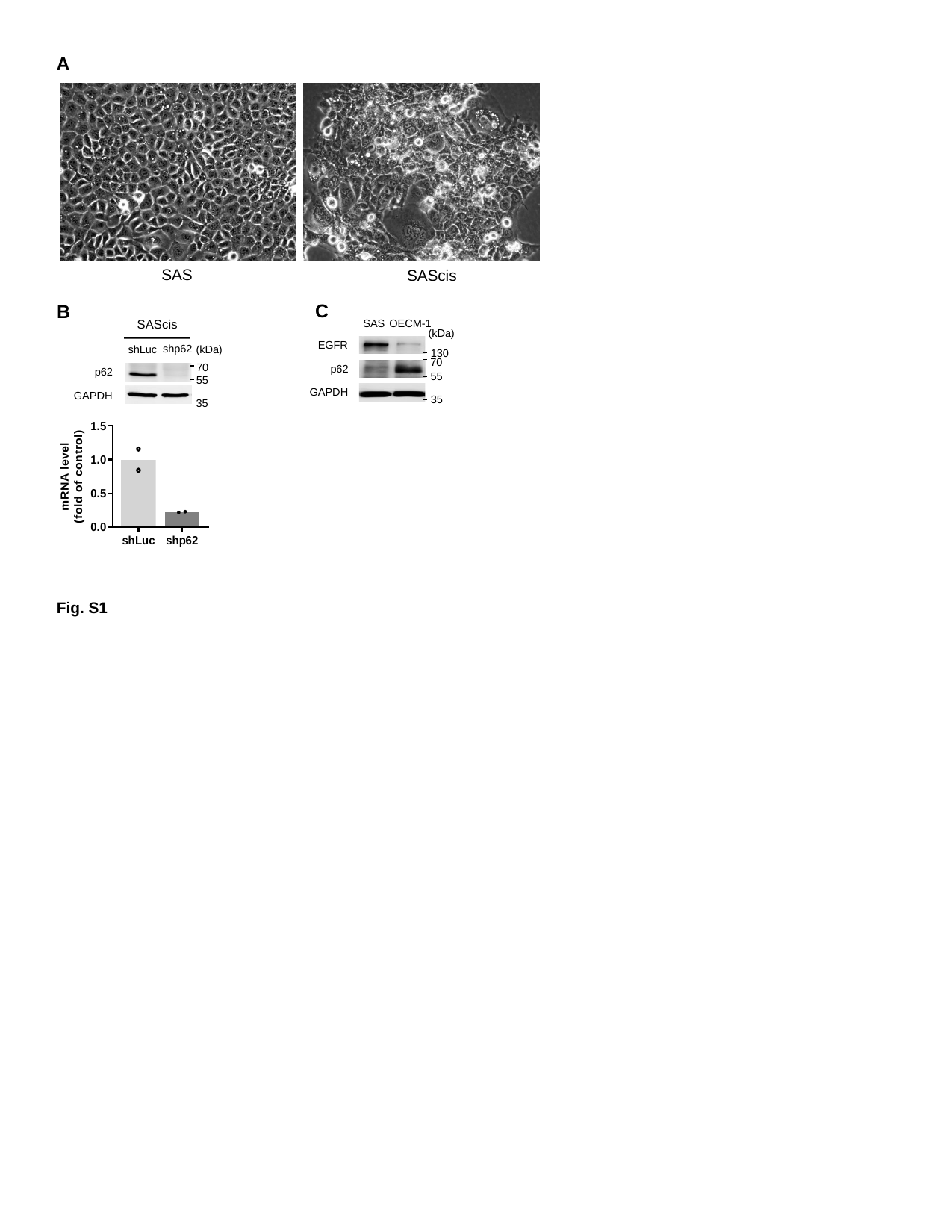

A
SAS
SAScis
C
B
OECM-1
SAS
(kDa)
EGFR
130
70
p62
55
GAPDH
35
SAScis
shp62
shLuc
(kDa)
p62
70
55
GAPDH
35
Fig. S1

Supplement: Multimedia component 1 [file mmc1.pptx]

## Slide 1
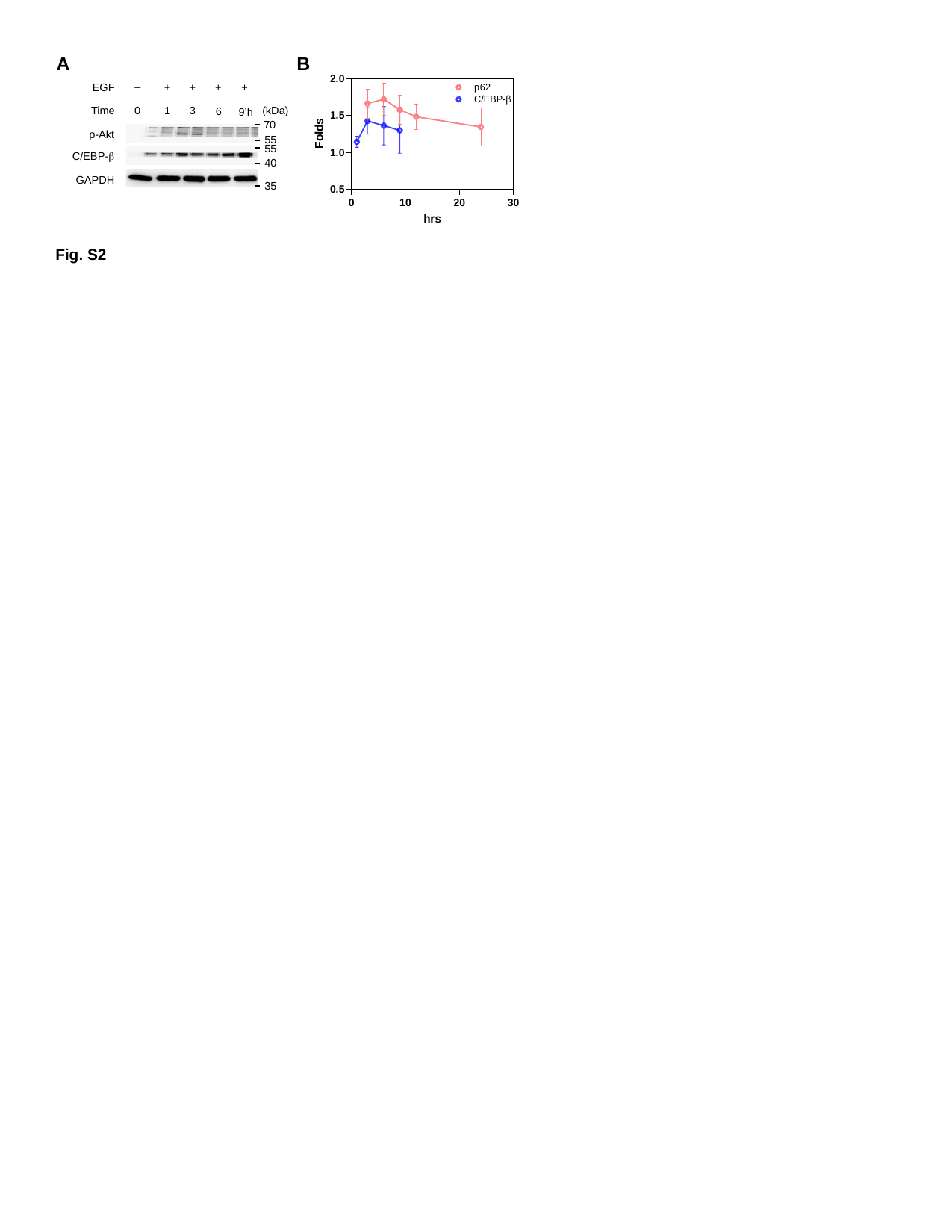

B
A
–
EGF
+
+
+
+
Time
(kDa)
0
3
1
6
9’h
70
p-Akt
55
55
C/EBP-β
40
GAPDH
35
Fig. S2

Supplement: Multimedia component 2 [file mmc2.pptx]

## Slide 1
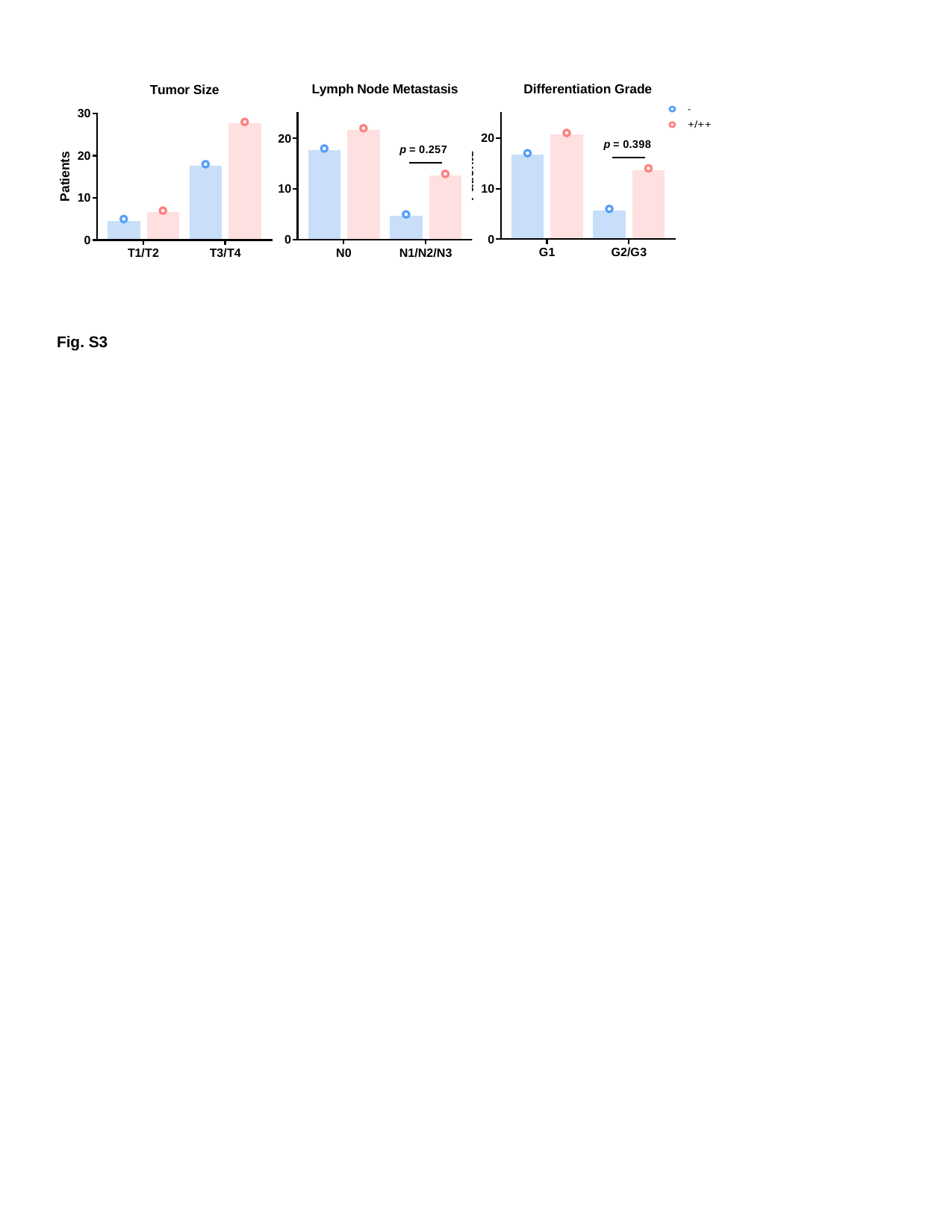

Fig. S3

Supplement: Multimedia component 3 [file mmc3.pptx]
