# Supplementary material for: Interplay of p62-mTORC1 and EGFR signaling promotes cisplatin resistance in oral cancer
Source: Heliyon. 2024 Mar 21;10(6):e28406. doi: 10.1016/j.heliyon.2024.e28406 (PMC10979205; doi:10.1016/j.heliyon.2024.e28406)
Supplement: Multimedia component 4 [file mmc4.docx]

Supplementary Tables

Table S1. Cell cultivation conditions

| **Cell line** | **Culture medium** | **Supplements** |
| --- | --- | --- |
| SAS | DMEM | 10% (v/v) FBS, 2 mM L-gultamine, 1% (v/v) penicillin-streptomycin-amphotericin B solution |
| OECM-1 | RPMI1640 | 10% (v/v) FBS, 1% (v/v) penicillin-streptomycin-amphotericin B solution |
| OC4 | DMEM/F12 | 10% (v/v) FBS, 1% (v/v) penicillin-streptomycin-amphotericin B solution |
| FaDu | MEM | 10% (v/v) FBS, 2 mM L-glutamine, 1 mM sodium pyruvate, 1% (v/v) penicillin-streptomycin-amphotericin B solution |
| HSC3 | DMEM/F12 | 10% (v/v) FBS, 1% (v/v) penicillin-streptomycin-amphotericin B solution |

1. The penicillin-streptomycin-amphotericin B solution, which contains penicillin G sodium salt (10,000 units mL^-1^), streptomycin sulfate (10 mg mL^-1^), and amphotericin B (25 µg mL^-1^), was purchased from Biological Industries (USA).
2. Other specified reagents were purchased from Gibco (USA).

Table S2. Antibodies used in the current study

| **Antibody** | **MW (kDa)** | **Species** | **Dilution** | **Supplier** | **Cat. No.** |
| --- | --- | --- | --- | --- | --- |
| p62/SQSTM1 | 65 | Mouse | 1:1000 | Santa Cruz Biotech | SC-28359 |
| p-mTOR (Ser2448)* | 289 | Rabbit | 1:50*  1:1000 | Cell Signaling Tech | 2976 |
| mTOR | 289 | Rabbit | 1:1000 | Cell Signaling Tech | 2983 |
| p-S6K (Thr389) | 70, 85 | Rabbit | 1:1000 | Cell Signaling Tech | 9205 |
| S6K | 70, 85 | Rabbit | 1:1000 | Cell Signaling Tech | 9202 |
| p-S6 (Ser235/236) | 32 | Rabbit | 1:2000 | Cell Signaling Tech | 4858 |
| S6 | 32 | Rabbit | 1:2000 | Cell Signaling Tech | 2217 |
| p-Akt (Ser473) | 60 | Mouse | 1:1000 | Cell Signaling Tech | 4051 |
| C/EBP-β | 40 | Mouse | 1:1000 | Santa Cruz Biotech | SC-7962 |
| Oct4 | 45 | Rabbit | 1:1000 | Cell Signaling Tech | 2750 |
| Nanog | 42 | Rabbit | 1:1000 | Cell Signaling Tech | 3580 |
| Actin | 43 | Mouse | 1:5000 | Merck Millipore | MAB1501 |
| GAPDH | 37 | Mouse | 1:1000 | Santa Cruz Biotech | SC-32233 |
| IgG |  | Rabbit | 2 μg | Sigma-Aldrich | I5006 |
| anti-Mouse IgG |  | Goat | 1:1000 | Merck Millipore | AP124P |
| anti-Rabbit IgG |  | Goat | 1:1000 | Merck Millipore | AP132P |

* IHC; other, Western blot analysis

Table S3. TaqMan^®^ probes (FAM) used in the current study

| **Gene** | **Cat. No.** |
| --- | --- |
| p62/SQSTM1 | Hs00177654_m1 |
| Oct4 | Hs01654807_s1 |
| Nanog | Hs04260366_g1 |
| Sox2 | Hs01053049_s1 |
| GAPDH | Hs00266705_g1 |

Table S4. Clinical Characteristics of the the patient subjects

| **Characteristic** | **OSCC patient** |
| --- | --- |
| Gender |  |
| Male | 51 |
| Female | 7 |
|  |  |
| Age |  |
| Mean±SEM | 56.34±1.65 |
| Range | 32-87 |
|  |  |
| Tumor size |  |
| T1/T2 | 12 |
| T3/T4 | 46 |
|  |  |
| Lymph node metastasis |  |
| N0 | 40 |
| N1/N2/N3 | 18 |
|  |  |
| Adjuvant therapy |  |
| No | 30 |
| Yes | 28 |
|  |  |
| Recurrence |  |
| No | 41 |
| Yes | 17 |
